# The DSM-5 criteria, level of arousal and delirium diagnosis: inclusiveness is safer

**DOI:** 10.1186/s12916-014-0141-2

**Published:** 2014-09-25

**Authors:** European Delirium Association, American Delirium Society

**Affiliations:** MRC Unit for Lifelong Health and Ageing, University College London, 33 Bedford Place, London, WC1B 5JU UK

**Keywords:** Delirium, Consciousness, Arousal, Attention, Diagnostic and Statistical Manual of Mental Disorders

## Abstract

**Background:**

Delirium is a common and serious problem among acutely unwell persons. Alhough linked to higher rates of mortality, institutionalisation and dementia, it remains underdiagnosed. Careful consideration of its phenomenology is warranted to improve detection and therefore mitigate some of its clinical impact. The publication of the fifth edition of the *Diagnostic and Statistical Manual* of the American Psychiatric Association (DSM-5) provides an opportunity to examine the constructs underlying delirium as a clinical entity.

**Discussion:**

Altered consciousness has been regarded as a core feature of delirium; the fact that consciousness itself should be physiologically disrupted due to acute illness attests to its clinical urgency. DSM-5 now operationalises ‘consciousness*’* as ‘changes in attention*’*. It should be recognised that attention relates to content of consciousness, but arousal corresponds to level of consciousness. Reduced arousal is also associated with adverse outcomes. Attention and arousal are hierarchically related; level of arousal must be sufficient before attention can be reasonably tested.

**Summary:**

Our conceptualisation of delirium must extend beyond what can be assessed through cognitive testing (attention) and accept that altered arousal is fundamental. Understanding the DSM-5 criteria explicitly in this way offers the most inclusive and clinically safe interpretation.

## Background

Delirium is an extensive and serious problem in acute hospitals [1]. It is unquestionably a marker for vulnerability, and is associated with adverse outcomes in a number of settings [2-5]. Fundamentally, the syndrome represents a decompensation of cerebral function in response to one or more pathophysiological stressors [6]. Therefore, understanding how to identify delirium can be central to recognising acute illness in patients of all ages. The American Psychiatric Association’s fifth edition of the *Diagnostic and Statistical Manual of Mental Disorders* (DSM-5) revised the diagnostic criteria for delirium. As the leading organisations in delirium science and practice, the European Delirium Association (EDA) and American Delirium Society (ADS) believe that the interpretation of these revisions warrants comment, in order to improve clinical practice and patient safety.

The diagnosis of delirium represents an umbrella construct that was adopted to overcome the terminological chaos existing before DSM-III (1980), when dozens of terms were used to indicate generalised brain dysfunction occurring in the context of acute illness or drug intoxication. These included *‘acute confusional state’, ‘encephalopathy’, ‘acute brain failure’, ‘ICU psychosis’, and even ‘subacute befuddlement’* [7,8]. These terms were not based upon any explicit scientific rationale, but rather denoted delirium occurring in different patient populations and/or treatment settings. Combining all of these clinical constructs under the term *‘*delirium’ has resulted in a more coherent approach to clinical practice and research.

A consistent feature of DSM versions prior to DSM-5 has been the requirement that alterations in the content (that is, attention) and/or level (that is, arousal) of consciousness are core to the diagnosis of delirium. Delirium can present as hypoactive or hyperactive states, and may fluctuate between the two. DSM-III used the term *‘*clouding of consciousness’. DSM-III-R and DSM-IV, while maintaining the term *‘*consciousness’, operationalised this by linking this construct to deficits in attention. This shift towards attention was driven by a recognition that the construct ‘consciousness*’* was difficult to assess objectively [9]. It should be appreciated that for consciousness, both attention and arousal are hierarchically related: it is possible to have full arousal, but profound inattention (for example, hypervigilance), but not the other way around [10]. Therefore, the retention of ‘consciousness*’* implied that level of arousal remained part of the construct of delirium.

In DSM-5, the term ‘consciousness’ is not used at all (Table 1). Delirium is now more restrictively defined in terms of its cognitive features, and the level of arousal element implicit in prior DSM criteria has been removed. Moreover, Criterion D states that inattention or changes in cognition ‘must not occur in the context of a severely reduced level of arousal such as coma’.

**Table 1:** **Comparing DSM classifications of delirium** ^**a**^

**DSM-5**	**DSM-IV**
A. Disturbance in *attention* (i.e., reduced ability to direct, focus, sustain, and shift attention) and awareness (reduced *orientation to the environment*).	A. Disturbance of consciousness (i.e. reduced clarity of awareness of the environment) with reduced ability to focus, sustain or shift attention.
B. The disturbance develops over a short period of time (usually hours to a few days), *represents an acute change from baseline attention and awareness*, and tends to fluctuate in severity during the course of a day.	B. A change in cognition or the development of a perceptual disturbance that is not better accounted for by a pre-existing, established or evolving dementia.
C. An additional disturbance in cognition (e.g.memory deficit, disorientation, language, visuospatial ability, or perception).	C. The disturbance develops over a short period of time (usually hours to days) and tends to fluctuate during the course of the day
*D.* * The disturbances in Criteria A and C* are not better explained by a pre-existing, established or evolving neurocognitive disorder and *do not occur in the context of a severely reduced level of arousal such as coma.*	D. There is evidence from the history, physical examination or laboratory findings that the disturbance is caused by the direct physiological consequences of a general medical condition.
E. There is evidence from the history, physical examination or laboratory findings that the disturbance is *a direct* physiological consequence of another medical condition, *substance intoxication or withdrawal (i.e. due to a drug of abuse or to a medication), or exposure to a toxin, or is due to multiple etiologies*.	

DSM-IV, *Diagnostic and Statistical Manual of Mental Disorders*, fourth edition; DSM-5, *Diagnostic and Statistical Manual of Mental Disorders*, fifth edition.

^a^Changes in DSM-5 from DSM-IV shown in *italics*.

## Discussion

**Figure 1:**
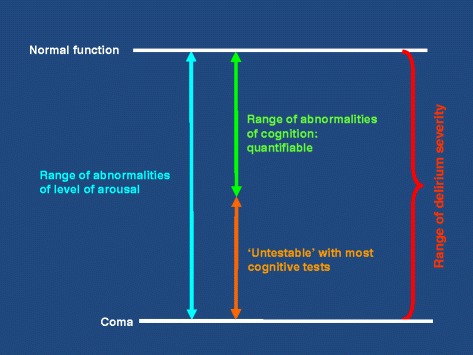
**Overlap between hypoactive delirium and reduced arousal states (hyperactive delirium not included).**

The risk of misinterpreting these revised criteria is that clinicians may focus inappropriately on inattention and testability, erroneously overlooking the *de facto* disturbance in consciousness (that is, delirium) that comes with altered arousal. Criterion D draws attention to the idea that altered arousal states may exist outside of delirium. Our view is that this is only the case in the profoundest possible disturbance of arousal, namely, coma (Figure 1). Other than coma, the interpretation of Criterion D should recognise that it is not possible to determine a threshold to discriminate severe and non-severe levels of arousal. It is also worth noting, in relation to Criterion E, that withdrawal of an antipsychotic in a patient with a chronic psychotic condition, such as schizophrenia, may result in a syndrome of increased arousal and acute recurrence of psychotic symptoms. Although this specific state may appear to be phenomenologically similar to delirium, it should not be classified as such.

What kinds of evidence should be required to demonstrate disturbances in attention, orientation and other cognitive domains? A narrow interpretation of Criterion D could mean that patients too drowsy to undergo cognitive testing cannot fulfil Criterion A (inattention and disturbed orientation to the environment) or Criterion C (deficit in an additional cognitive domain). That is, patients not capable of demonstrating ‘inattention’ cannot be assessed against Criterion A if this is interpreted to mean that patients must show impaired performance on cognitive tests of attention or an inability to sustain attention during interview. Of crucial clinical importance, non-comatose patients who are too drowsy to demonstrate inattention by tests or interview might not be classified as having delirium. This narrow approach would have multiple negative consequences. The unanimous view of the Boards of the EDA and ADS is that Criterion D should include all states of altered arousal (except coma) in the spectrum of delirium on scientific, practical and clinical safety grounds.

First, a substantial proportion of patients present to acute hospitals with reduced consciousness that is severe enough to affect their ability to engage with cognitive testing and/or interview. Reduced level of consciousness is present at least 8% of general hospital admissions [11]. If Criterion D is strictly applied, large numbers of patients will thus be left unclassified, or labelled with vague descriptions such as *‘*obtunded’ *or* ‘stuporose*’.* This is important, because reduced level of arousal is a powerful predictor in early warning scores of mortality [11] and subsequent admission to intensive care [12]. The clinical approach to such patients is essentially the same as the approach to verbally communicative patients with delirium. Access to delirium management pathways, present in increasing numbers of hospitals, is beneficial, and ambiguity about which non-comatose but acutely mentally impaired patients undergo such pathways will likely lead to worse care for some.

Second, there is no clear empirical evidence that non-comatose patients who are verbally uncommunicative are different from patients with milder degrees of arousal impairment in whom inattention can readily be demonstrated through verbal responses. Evidence from animal and human studies suggests that there is a continuum of levels of arousal. The little direct empirical evidence that exists in humans actually suggests that reduced arousal is highly specific for delirium [13].

Third, segmenting the spectrum of acutely reduced arousal into ‘delirium’, ‘other’, and ‘coma’ would present substantial difficulties to both clinicians and researchers. In non-comatose patients with acutely reduced level of arousal that is severe enough for them to be unable to engage verbally, the clinical approach is essentially the same as in patients who are well enough to communicate. Therefore, it is not rational to divide the non-coma part of the spectrum. Moreover, fluctuations in level of arousal mean that patients could have a diagnosis of delirium in one part of the day, but then lose this diagnosis (and enter a vaguely defined category) if they later became so drowsy that they were unable to communicate verbally. This degree of fluctuation is observed frequently by clinicians. Interpreting the criteria in such a way that only part of the spectrum is covered, especially when patients are fluctuating along this spectrum, is impractical.

## Summary

To conclude, an inclusive interpretation of Criteria A and D is essential. Patients who are not comatose, but have impaired arousal resulting in an inability to engage in cognitive testing or interview (for example, drowsiness, obtundation, stupor or agitation), must be understood as effectively having inattention. Including such patients under the umbrella of delirium is more closely aligned with the scientific evidence and the realities of clinical practice, and will result in increased patient safety through broader delirium prevention and identification.

## Abbreviations

ADS: American Delirium Society
DSM: *Diagnostic and Statistical Manual of Mental Disorders*
EDA: European Delirium Association
